# Deep Learning Approaches for Classifying Children With and Without Autism Spectrum Disorder Using Inertial Measurement Unit Hand Tracking Data: Comparative Study

**DOI:** 10.2196/73440

**Published:** 2025-12-22

**Authors:** John Mutersbaugh, Wan-Chun Su, Anjana Bhat, Amir Gandjbakhche

**Affiliations:** 1 NICHD, National Institutes of Health, NIHBC 49 - Conte 5A82, Bethesda, MD, 20892-4480, United States, 1 301-435-9235; 2 Louisiana State University, Baton Rouge, LA, United States; 3 University of Delaware, Newark, DE, United States; 4 Biomechanics and Movement Science Program, Physical Therapy Department, University of Delaware, , Newark, DE, United States

**Keywords:** AI, artificial intelligence, autism spectrum disorder, ASD, deep learning, kinematics, machine learning

## Abstract

**Background:**

Autism spectrum disorder (ASD) is a prevalent neurodevelopmental condition that can be quite difficult to diagnose due to a lack of objective diagnostic methods in the currently used behavioral assessments. Recent work has shown that children with ASD have a higher incidence of motor control differences. A compilation of studies indicates that between 50% and 88% of the children with ASD have issues with movement control based on standardized motor assessments or parent-reported questionnaires.

**Objective:**

In this study, we assess a variety of deep learning approaches for the classification of ASD, utilizing data collected via inertial measurement unit (IMU) hand tracking during goal-directed arm movements.

**Methods:**

IMU hand tracking data were recorded from 41 school-aged children both with and without an ASD diagnosis to track their arm movements during a reach-to-clean up task. The IMU data were then preprocessed using a moving average and *z* score normalization to prepare the data for deep learning models. We evaluated the effectiveness of different deep learning models using the preprocessed data and a k-fold validation approach, as well as a patient-separated approach.

**Results:**

The best result was achieved with a convolutional autoencoder combined with long short-term memory layers, reaching an accuracy of 90.21% and an *F*_1_-score of 90.02%. Once the convolutional autoencoder+long short-term memory was determined to be the most effective model for this datatype, it was retrained and evaluated with a patient-separated dataset to assess the generalization capability of the model, achieving an accuracy of 91.87% and an *F*_1_-score of 93.66%.

**Conclusions:**

Our deep learning approach demonstrates that our models hold potential for facilitating ASD diagnosis in clinical settings. This work validates that there are significant differences between the physical movements of typically developing children and children with ASD, and these differences can be identified by analyzing hand-eye coordination skills. Additionally, we have validated that small-scale models can still achieve a high accuracy and good generalization when classifying medical data, opening the door for future research into diagnostic models that may not require massive amounts of data.

## Introduction

Throughout the world, autism spectrum disorder (ASD) is a prevalent neurodevelopmental condition marked by challenges with communication in social settings, as well as restricted and repetitive behaviors [1,2]. An individual with autism spectrum disorder can have a range of challenges, primarily in social communication and interaction; for example, they may have difficulties understanding and responding to social cues, forming relationships, and effectively communicating with others using verbal and non-verbal languages [3-5]. Additionally, individuals with ASD may engage in repetitive behaviors or have highly specific interests, leading to an inflexibility in daily life activities and hardship when trying to keep up with fluid or dynamic social situations [6]. Behavioral patterns and communication difficulties like these tend to vary widely in presentation and severity, making ASD particularly challenging to diagnose. The most current methodologies for diagnosing ASD predominantly rely on behavioral evaluations and structured interviews conducted by trained clinicians [7,8]. An ASD assessment typically involves gathering a detailed history, conducting interviews with friends and family members, and sometimes observing an individual’s behavior in various social situations. However, despite the expertise of the clinicians involved, diagnosing an individual with ASD is very imprecise, depending heavily on the clinician’s subjective interpretation and experience [9]. The subjective nature of this diagnosis can be a problem; if different evaluators can come to different conclusions based on the same observations, then the chance for a missed diagnosis or a misdiagnosis is significant. This subjectivity also means that an ASD diagnosis might not properly reflect the subtleties of an individual, as ASD manifests a very wide spectrum of possible symptoms.

It is also worth noting that diagnosing ASD earlier in life is important; early access to support can significantly improve long-term outcomes and quality of life when addressed before behavioral patterns are solidly set [10]. Unfortunately, diagnosing ASD is much more difficult in young children, for all of the reasons explained before. For example, it is very subjective whether a child is having social communication problems or just being shy. Due to these challenges, there is a significant need for a more objective approach to diagnosing ASD.

Recently, it has been noted that there is a high incidence of motor control differences in children affected with ASD [11]. A compilation of studies indicates that between 50% and 88% of the children with ASD have issues with movement control based on standardized motor assessments or parent-reported questionnaires [11,12]. These children often exhibit significant differences with posture, hand-eye coordination, fine motor control, and gait. Importantly, these motor abnormalities are highly correlated with their primary social communication problems and manifest earlier or at the same time as any social communication issues [13-16]. This provides clinicians a new avenue for identifying ASD earlier.

In the past, it has been shown that machine learning and deep learning methods have been successful in classifying between children with ASD and typically developing (TD) children based on resting state functional magnetic resonance imaging data [17,18]. One study showed that a combination of a restricted Boltzmann machine and a support vector machine was able to achieve an accuracy of ~80% on a dataset of ~180 patients [18]. Another study using a deep learning model matched these results with a significantly larger dataset, achieving an accuracy of ~80% for a model classifying 871 participants [17]. Many other studies have been conducted attempting the same classification, using different combinations of functional near-infrared spectroscopy, magnetic resonance imaging, functional magnetic resonance imaging, and electroencephalogram data [19]. The average accuracies for these models are around 70%‐85%, with some specific models approaching 90%‐95%. The best functioning models that surpassed 90% accuracy were deep learning autoencoders, as well as multidimensional convolutional neural networks [19].

Despite the promising findings of using neuroimaging data to classify children with ASD, there are certain limitations. For example, neuroimaging is often expensive and difficult for typical families to access. Moreover, some neuroimaging tools are not child-friendly, making their widespread use in toddlers and children challenging. As a result, there is a pressing need for low-cost, child-friendly, objective methods to children’s performance. To solve these problems, we decided to use an inertial measurement unit (IMU) device. An IMU device is a very small motion–tracking device, easily strapped on to a participant’s wrist like a light-weight watch. These devices are nonintrusive and very discrete, allowing for data collection from sensitive children. Previously, our research group had achieved an accuracy of 78.1% in classifying children with and without ASD using IMU data and a multilayer perceptron model [20]. However, it remains unclear which deep learning methods would produce the best classification results. Therefore, the goal of our study is to evaluate a variety of simple and customizable deep learning models, including long short-term memory (LSTM), multilayer perceptron (MLP), and convolutional neural networks (CNNs), for an automatic classification of children with ASD. Recent studies have highlighted the growing efficacy of deep learning techniques such as these, including LSTM-based models for advancing the predictive accuracy in medical and diagnostic contexts. We will first assess the effectiveness of different types and configurations of deep learning models on this type of IMU data. Once the best models are determined, we will test them for effectiveness on unseen patient data.

## Methods

### Study Methodology

A total of 41 children, both with and without ASD, participated in this study (mean age [SE]: TD group: 10.3 [0.8], 8 male participants and 7 female participants; ASD group: 10.3 [0.5], 21 male participants and 5 female participants). No significant differences in age or sex were observed between the TD and ASD groups (*P*=.05). Participants were recruited through fliers, phone calls, and online postings distributed to local schools and community centers, as well as through Simons Powering Autism Research participant research match service [21]. The inclusion criteria of the TD children were (1) age between 6 and 17 years, while the exclusion criteria included (1) having any neurological or developmental diagnosis or delays, (2) having a history of preterm birth or significant birth complications, and (3) having a family history of ASD. For children with ASD, the inclusion criteria were (1) age between 6 and 17 years and (2) having a professionally confirmed diagnosis, while the exclusion criteria were (1) inability to follow 1-step instruction; (2) exhibiting sensory and behavioral challenges that would prevent them from wearing the IMU sensors and completing the reaching-and-placing tasks. These criteria were chosen for a very simple reason: if a child has ASD so severe it prevents them from engaging in the task, such as having issues wearing the hand device or following instructions, it is likely that they would not need this test to diagnose them in the first place. We are attempting to evaluate ASD based on significant differences in hand-eye coordination skills and approaches, so the TD children and the children with ASD were not screened for hand-eye coordination skills to ensure fair results. Our hypothesis is that a deep learning model can evaluate the harder-to-define edge cases of ASD by identifying key differences in how children with or without ASD interact with a simple hand-eye coordination task.

**Figure 1.**
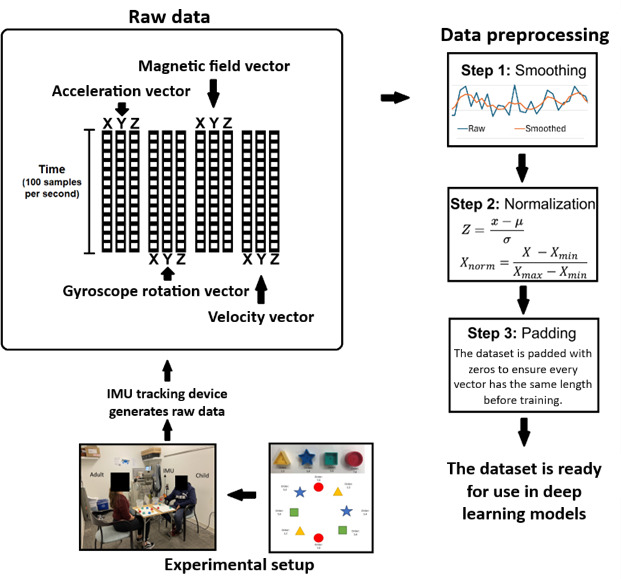
The experimental setup, consisting of an adult experimenter, a child participant wearing an inertial measurement unit (IMU) device, and a table with a series of blocks setup and a bucket next to the blocks. Also shown is the rough shape of the raw IMU data, as well as the data preprocessing steps performed to prepare the data for use in a model.

The hand-eye coordination task itself is simple; while seated across from an adult experimenter, each participating child performed a simple reaching task while wearing a small IMU hand tracking device fastened to the wrist of their right hand. The task itself consisted of a set of blocks arranged in a circle and placed in front of the participant, as shown in Figure 1. Each participant would pick up the blocks in a specific order, specified by the picture instructions, and place each block one at a time into a box next to them until all the blocks had been moved. This task was repeated for a total of roughly 6 trials per child, with each trial taking anywhere from ~10 seconds to ~40 seconds to complete. Once the data collection was complete, the final dataset consisted of 76 trials from TD children and 145 trials from children with ASD.

### Ethical Considerations

All study procedures were carried out in accordance with the Declaration of Helsinki and were approved by the University of Delaware Institutional Review Board. Informed consent from parents and assent from children were obtained prior to participation. Each participant was assigned a unique study ID, and all collected data were subsequently deidentified using these IDs. Families received US $20 as compensation for their participation. Additionally, written permission was obtained from parents and experimenters for the use of their photographs in this publication.

### Data Processing and Analysis

The primary outputs of the IMU device are the acceleration vector, the gyroscopic rotation vector, the velocity (Δv) vector, and the magnetic field vector, with each showing a basic representation of the movement in space. Each vector consists of an x, y, and z component to represent the movement of the IMU device in space, sampled at 100 Hz (Figure 1). The first 3 sets of movement vectors (acceleration, velocity, and rotation) are important for calculating the exact position of the IMU device in 3D space. Then, the magnetic data vector was used because the magnetic data are critical to counteract any drift experienced by the accelerometer and gyroscope. At this point, the raw data consisted of just these 4 vector types, with the final shape of the combined data being an Nx12 array, where the unknown length N is equivalent to the amount of time the participant took to complete the task in seconds, multiplied by 100 due to the sampling rate, as shown in Figure 1.

In order to prepare the data for use in a deep learning model, several preprocessing steps were implemented, as shown in Figure 1. First, a 3-point moving average was applied to each of the x, y, and z components of each vector to slightly smooth out the data without losing any of the features. Small-scale testing showed that a little bit of smoothing is critical to the accuracy of the model; it helps the model look for generalizations between different trials without getting caught up on outliers. Next, the data were normalized by applying a global *z* score, followed by a global min-maxing. A *z* score is an algorithm to normalize a signal of any length based on the mean and SD, and it has been shown to significantly improve model accuracy and generalization capability [22]. By applying the *z* score function globally, it standardizes the data by bringing all values into the same reference frame, relative to their mean and SD. Min-maxing is not strictly necessary after a *z* score, but it has also been found to improve model accuracy [22]. It does this by ensuring that all values fall between 0 and 1, preventing deep learning models from experiencing runaway gradients and thus speeding up convergence during training. Finally, the trials with different lengths were zero-padded to be equal length, producing data in the shape 4000×12. While a few specific types of deep learning can handle data of different lengths during training, most of the model architecture we will be using is too rigid to handle variable length data, requiring a fixed input size.

After determining the best preprocessing steps for the data, a few final concerns need to be addressed. One concern is the variable length of the data; if the ASD class contains many trials that are significantly longer or shorter than the TD trials, the model could just end up classifying based on length rather than any feature characteristics (features) of children with ASD. It is generally expected that roughly 95% of the data in a normal distribution would fall within 2 SDs of the mean, and beyond this, the data might be considered unusual or even an outlier. Checking the dataset, the average length of a trial is roughly 1897 samples, with an SD of 463. The entire set of TD trials falls within 2 SDs of the mean, and only 13 trials of 145 from the children with ASD dataset are outside 2 SDs, all of them longer. This is a fairly normal distribution of data, where roughly 94% of the data are within 2 SDs of the mean. It is not quite normal to have every trial beyond 2 SDs on one side of the distribution curve, but it was decided that none of the data would be excluded as outliers because a slightly longer trial time might be a characteristic of children with ASD. Also, 13 of the 221 trials being significantly longer would not overwhelm the model, meaning that to achieve any real accuracy, it would have to learn other real characteristics of children with ASD from the kinematics data. After this, 1 final issue needed to be considered. The dataset consisted of 76 trials from TD children and 145 trials from children with ASD, meaning 1 possible classification had nearly double the amount of data compared to the other classification. Generally, deep learning models function best with balanced datasets, meaning they need a similar amount of data from all possible classifications; otherwise, they can run into major training issues. To solve this problem, a simple data balancing technique was employed. The smaller TD dataset was doubled, for a final dataset of 152 TD trials and 145 ASD trials. This data balancing method allows us to compare the validation accuracies, which we used to prototype several kinds of models. This allowed us to determine which models work best on this kind of data, and which models do not function at all.

With the data preprocessed, the next step was to prototype and test several deep learning models. Before any specific model methodologies could be compared, it is critical to determine the basic parameters that will ensure all the models are functioning as best as they can. Parameters such as the number of layers, the number of neurons in the layers, the learning rate, the best activation functions, the overall length of time required to train the model, and the video random-access memory (VRAM) requirements of the models were refined after running the tests shown in Table 1. It should be noted that while almost all of the above parameters are critical for ensuring peak functionality of the models, the training time and VRAM measures are simply a limitation of our computing resources for building and testing models. All models were trained with the Adam optimization algorithm and tested using a k-fold cross-validation scheme.

### Video random-access memory

The requirements of the models were refined after running the tests shown in Table 1. It should be noted that while almost all of the above parameters are critical for ensuring peak functionality of the models, the training time and VRAM measures are simply a limitation of our computing resources for building and testing models. All models were trained with the Adam optimization algorithm and tested using a k-fold cross-validation scheme.

**Table 1.** Initial testing results from our model prototyping^a^.

Model prototype	Cross-validation accuracy (%)	Training time for 1 k-fold (h)
LSTM^b^ only, 1 layer, 3×12 neurons	57.59	~3
LSTM only, 5 layers, 3×12 neurons	62.76	~6
LSTM only, 1 layer, 6×12 neurons	66.21	~8
LSTM only, 5 layers, 6×12 neurons	67.59	~18
LSTM only, 1 layer, 12×12 neurons	65.17	~33
LSTM only, 5 layers, 12×12 neurons	71.03	~72
MLP^c^ only, 1 layer	89.31	~1
MLP only, 3 layers	92.21	~1.5
MLP only, 6 layers	88.96	~2
MLP only, 9 layers	83.41	~3.5
CNN^d^ only, 3 layers	90.34	~2
CNN only, 5 layers	91.38	~3
CNN only, 10 layers	87.14	~4
Transformer, 8 heads, 4 layers	82.73	~20

a Several versions of the long short-term memory models, multilayer perceptron models, convolutional neural network models, and transformer models were tested. All of these tests were performed using the balanced dataset mentioned previously. Because of this, these results do not represent the effectiveness of our motor task for diagnosing ASD, only the effectiveness of the models classifying this type of IMU data.

b LSTM: long short-term memory.

c MLP: multilayer perceptron.

d CNN: convolutional neural network.

Several key design choices were based on the model prototyping data collected. First, it was decided that any models with LSTM layers attached would use the 1 layer, 6×12 neuron format, as this seemed to provide the best possible LSTM classification, while keeping the runtime and VRAM requirements at a reasonable level. As Table 1 shows, models with significantly more LSTM neurons increase the training time to a matter of days to weeks, which would not be feasible for running many tests on many model permutations.

The second design choice implemented after testing was the use of only 3 layers for all future MLP testing. While adding more MLP layers does not increase the training time like LSTM layers do, having too many layers with too many neurons causes major overfitting problems, leading to the brute force memorization of the training data rather than any real generalizations.

It was also decided that all non-MLP models would have a smaller 3-layer MLP attached to the end as the final classifier. This allows the models a way to concatenate the output from CNN layers with the output from LSTM layers. It also has the side benefit of making all the models directly comparable; by keeping the final classification portion of the models the same, any changes in the features extracted by the LSTM, CNN, and convolutional autoencoder (CNN-AE) model configurations will be apparent. Thus, all models were designed to have 3 MLP layers attached at the end as the final classifier.

Other key design choices informed by the data from Table 1 include limiting the number of CNN layers to 5 to prevent overfitting, using the LeakyReLU activation functions on all layers except for the output layer, using the sigmoid activation function for the output layer, using a learning rate of 1e-4, and training for 200 epochs. Also worth noting is that several transformer type models were tested with no success, likely due to the limited amount of VRAM available for training these models. No further improvements were possible for the transformer type models with our training setup, so this model type was not evaluated any further. Now that the main model parameters have been identified, 6 types of deep learning models were built to test their effectiveness on this task and data type.

**Figure 2.**
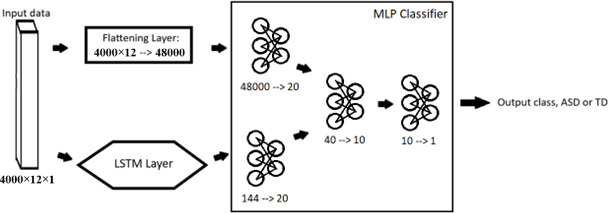
Schematic for the first merged model tested: the merging of a multilayer perceptron (MLP) classifier and a long short-term memory (LSTM) classifier. The MLP-only model takes the same form as this model, minus all of the LSTM-specific layers. ASD: autism spectrum disorder; TD: typically developing.

The MLP type models (Figure 2) each had 3 primary linear layers, with a batch normalization layer between each layer. LeakyReLU activate functions were used for the first 2 layers, and the third layer used a sigmoid function for the classification output. Before the first layer, there is a squeeze function to transform the 4000×12 zero-padded data input into a vector of length 48,000, ready for use in the MLP layers. We found that the size of the first layer was quite important; using an output value of much larger than 20 would cause significant overfitting, and using an output much smaller would cause the model training time to increase significantly, by requiring hundreds to thousands more epochs to reach the same level of accuracy. The key difference between the MLP and the MLP+LSTM is the addition of an LSTM layer along with an extra linear layer, as shown in Figure 2. The LSTM layer received the preprocessed input data as a sequence, and the cell state and hidden state were used as its output. Critically, the LSTM layers are able to handle data with differing input sizes, so the zero-padding on the input data was stripped before being input into the LSTM layers. The output from the LSTM was then fed into a dedicated linear layer to prune the features and produce a separate output of length 20. This 20-point output was then concatenated with the 20-point output produced from the first layer as shown above, and the merged data were then run through the final 2 layers to produce a single classification. Just like the MLP model, the MLP+LSTM used batch normalization between all layers and LeakyReLU for all layers except for the classification, which used the sigmoid function (Textbox 1).

Textbox 1. **Six final model architectures that have been evaluated. There are 3 primary model designs to be tested, the multilayer perceptron (MLP), convolutional neural network (CNN), and convolutional autoencoder (CNN-AE), and a modified version of each of these 3 models using long short-term memory (LSTM) layers. Each type of model was set up with matching layer structures to allow for a more direct comparison of effectiveness.**

**Deep learning models to be evaluated**

- MLP
- CNN
- CNN-AE
- MLP+LSTM
- CNN+LSTM
- CNN-AE+LSTM

**Figure 3.**
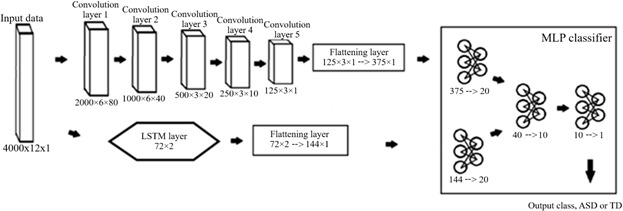
Schematic for the merged convolutional neural network with long short-term memory layers (CNN+LSTM) model. The CNN-only model takes the same form as this model, minus all of the LSTM-specific layers. ASD: autism spectrum disorder; TD: typically developing.

The CNN and CNN+LSTM models used a somewhat similar setup to the MLP-only models, with 1 key difference. The CNN model starts with 5 convolutional layers, transforming the input from 4000×12 to 125×3×1, as shown in Figure 3. The first 2 convolutional layers use a window size of 5×5, with the last 3 layers using a window size of 3×3. The optimal sizes of the window in CNN type models can vary greatly from dataset to dataset; however, it has been shown that prime numbers can be very effective in the first 2 layers of a CNN, while the deep layers optimize efficiently when they are small [23]. The stride for each convolution was specifically tuned to slowly compress the featurespace between each convolution, while ensuring that the model does not require too much memory or take too much time to run. The channel count was also tuned for this purpose, while ensuring that the maximum amount of features can be extracted from the data. Each channel functions as a featuremap, a 2D grid that represents all features detected by the convolution window. Importantly, each channel has its own window with optimizable weights. This is why the channel count starts at maximum then slowly decreases layer by layer, to ensure that all possible features can be detected at first, and then refined into the most important set of features from the data. Next, a batch normalization was implemented between each convolutional layer. When tested without batch normalization, these models all take significantly more epochs to train to the same accuracy. This means that adding batch normalization helps these models converge significantly faster. Each convolution also used a LeakyReLU activation function. Afterward, the 125×3×1 vector was squeezed into a linear vector, for use in the final MLP classifier. These layers were set up the same as the MLP model from before, allowing for a direct comparison between the methods. Similarly, the CNN+LSTM model concatenates the results from the convolutional layers and the LSTM layer in this final MLP classifier.

**Figure 4.**
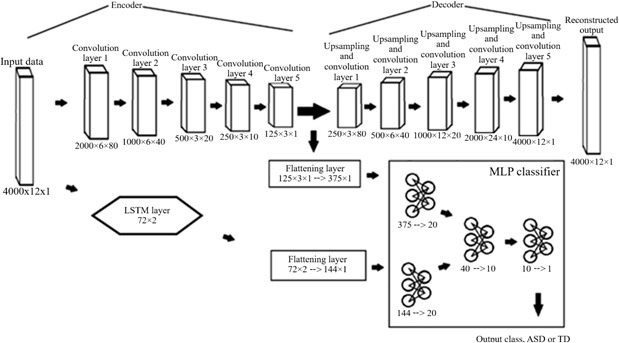
Schematic for the merged convolutional autoencoder with long short-term memory layers (CNN-AE+LSTM) model. The CNN-AE only model takes the same form as this model, minus all of the LSTM-specific layers. ASD: autism spectrum disorder; TD: typically developing.

The last kind of model we tested was a CNN-AE. This model functions differently from the previous 2 types we have discussed. An MLP model and a CNN model only function to classify the data they are trained on, while an autoencoder can both classify and reconstruct the data it has been trained on. Autoencoder reconstruction works by compressing the input data into a smaller featurespace with an encoder, then rebuilding the original input data back from the compressed featurespace with a decoder. This method of compressing and reconstructing forces the model to identify key signal features fundamental to its composition. In this case, the encoder section has the same convolutional layers as the previous CNN models, compressing the input data from 4000×12 to 125×3×1. The decoder then uses a series of upsampling layers combined with convolutional layers to reconstruct the signal back from 125×3×1 to 4000×12, as shown in Figure 4. Afterward, the model sends the compressed featurespace through linear layers to generate the final classification, using the same layer formatting as all the previous models. Just as with the previous models, the CNN-AE+LSTM model integrates the LSTM data with the featurespace during the same step.

All of these models were tested using k-fold cross-validation, where k was 10. This means that the data are split up into 10 random subgroups, the model is trained on 9 subgroups and tested on the 10th subgroup, and the resulting accuracy is saved for later. This process is repeated 9 more times, using each unique subgroup as the testing data one time while training on the rest. After all the training is done, all 10 accuracies are averaged to produce the final cross-validation accuracy.

## Results

**Figure 5.**
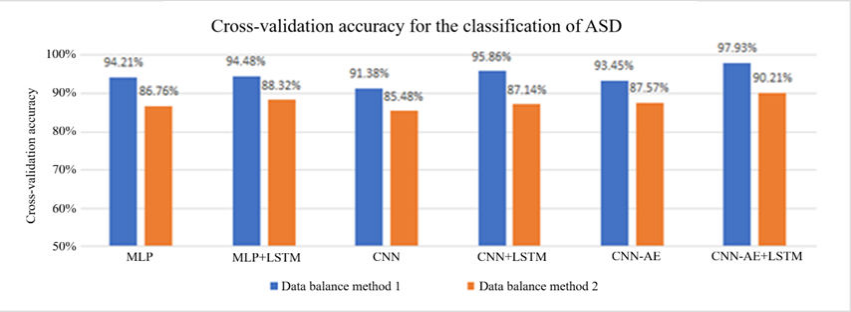
These are the resulting accuracies from the 2 training sessions for each model. Data balancing method 1 cannot be considered evidence for the effectiveness of the motor task due to the duplication of data in the training and testing sets. Data balancing method 2 is statistically valid and does show the effectiveness of our motor task for distinguishing between children with autism spectrum disorder (ASD) and typically developing (TD) children. CNN: convolutional neural network; CNN-AE: convolutional autoencoder; LSTM: long short-term memory layer; MLP: multilayer perceptron.

All 6 models were trained and evaluated for accuracy, with several important results. First, we noticed that using LSTM layers significantly increases the accuracy each model can achieve, as shown below by the blue bars in Figure 5. These data also show that the CNN-AE with added LSTM layers is the best model. Unfortunately, it was at this point that we discovered that these resulting accuracies (labeled in blue in Figure 5) were misleadingly high, due to a fault with the data balancing method. By simply duplicating the TD dataset, the same piece of data could end up in both the training and testing set, due to the random sampling of the data during the standard use of the k-fold cross-validation method. This means that while the model-to-model trend in the data is likely correct, the blue columns in Figure 5 are statistically invalid for drawing conclusions about the effectiveness of the cognitive-motor task.

Fortunately, there was a fairly simple way to fix this problem, allowing us to evaluate the effectiveness of our cognitive-motor task. A second data balancing method was implemented, where the larger dataset of ASD trials was split in half, creating one dataset that is 73 ASD trials and 76 TD trials, and another dataset with the remaining 72 ASD and the same 76 TD trials. Each model was evaluated using both datasets, and the cross-validation accuracies were averaged for the final resulting accuracy. This is a statistically useful test for evaluating the effectiveness of the cognitive-motor task, as it ensures that no datapoints from the training set are ever introduced into the testing set. Thus, the accuracy achieved by a model depends on its successful interpretation of the cognitive-motor task. The results from using this second method of data balancing are labeled in orange in Figure 5. These results allow us to assess how well our cognitive task works for the deep learning classification of a participant; clearly, the task is effective, as the average model accuracies range from ~85% to ~90%. Also clearly shown is how the second data balancing method produces the same trend in the data, with the CNN-AE+LSTM once again proving to be the best model.

While accuracy is a great general metric for evaluating the effectiveness of a deep learning model, it is not the only metric that should be considered. For starters, looking at the SD for the models can tell us approximately how reliable a model is, where a low standard deviation means a model is quite stable between k-folds. For our models using data balancing method 2, the average standard deviation was no greater than ~2%. This means our models are stable when they produce these accuracies, which is a good sign for possible future work. Stable models are more likely to generalize successfully when trained with larger datasets. Precision and recall are very useful scores for judging medical artificial intelligence (AI) performance, because these metrics are calculated using false-positive and false-negative results, respectively. When AI is being used as a diagnostic tool, false-positive diagnoses can lead to unnecessary treatment and wastage of time, while false-negative results can lead to a patient missing required care. Both false-positive and false-negative cases are critically important to avoid, so we calculated the *F*_1_-score for each of our models. The *F*_1_-score is a combination of precision and recall that can be considered more important than accuracy for evaluating our diagnostic model in this case. To calculate these scores, the models were run again to generate confusion matrices. The models were run with the second data balancing method described above, where the dataset was split in two, the models run twice, and the results averaged to produce the final scores.

**Figure 6.**
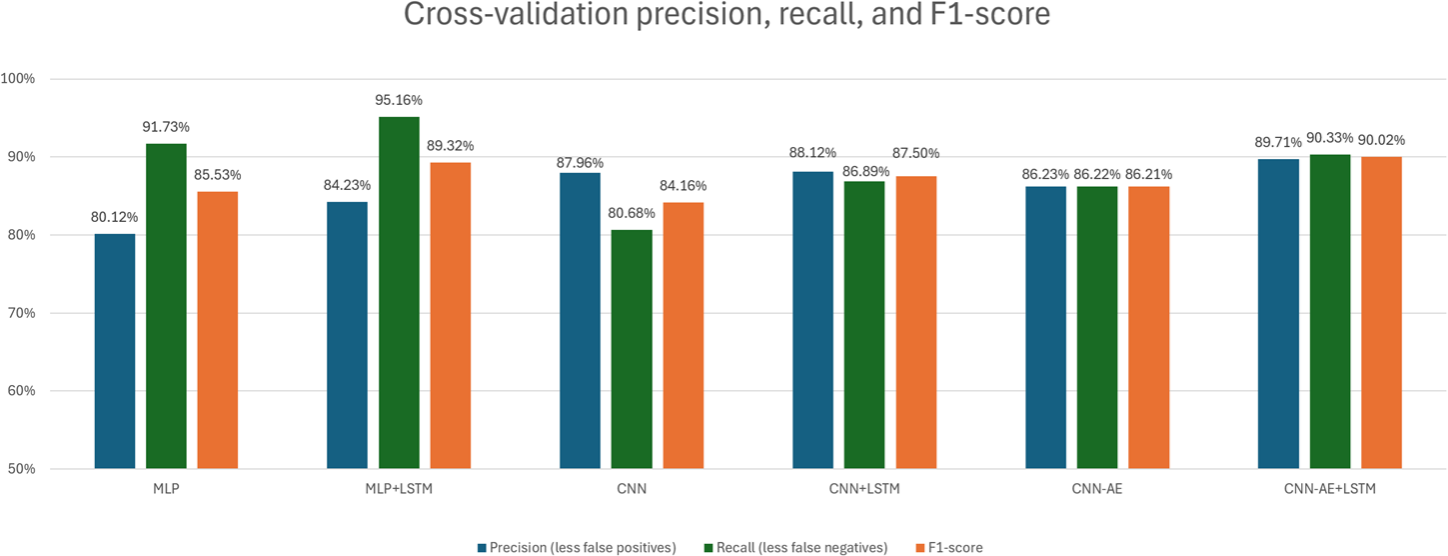
These are the precision, recall, and *F*_1_-scores for each model. The number above each set of columns is the *F*_1_-score for each model. All of these scores were calculated using the statistically valid second data balancing method mentioned previously. CNN: convolutional neural network; CNN-AE: convolutional autoencoder; LSTM: long short-term memory; MLP: multilayer perceptron.

Based on Figure 6, we see that the *F*_1_-scores match closely with the trend shown previously by the accuracies in Figure 5. Adding LSTM layers is once again shown to be vital to increasing the effectiveness of these models when predicting children with ASD. Interestingly, the MLP models score consistently higher recall values with corresponding lower precision values. The higher recall score means that the MLP type models might have less false-negative results overall, ensuring that participants with ASD will not be missed by the model. This does come with the downside of a lower precision, meaning that participants without ASD might be accidentally diagnosed positively more often. The CNN models seem to show the inverse, with higher precision and lower recall, but this might fall within the range of error, due to the random initializations of the weights whenever a new training session is started. Finally, we can see that the autoencoder models achieve a balance between precision and recall, with the LSTM autoencoder achieving a slightly higher *F*_1_-score of 90.02%, meaning this is a better overall optimization as a result.

**Figure 7.**
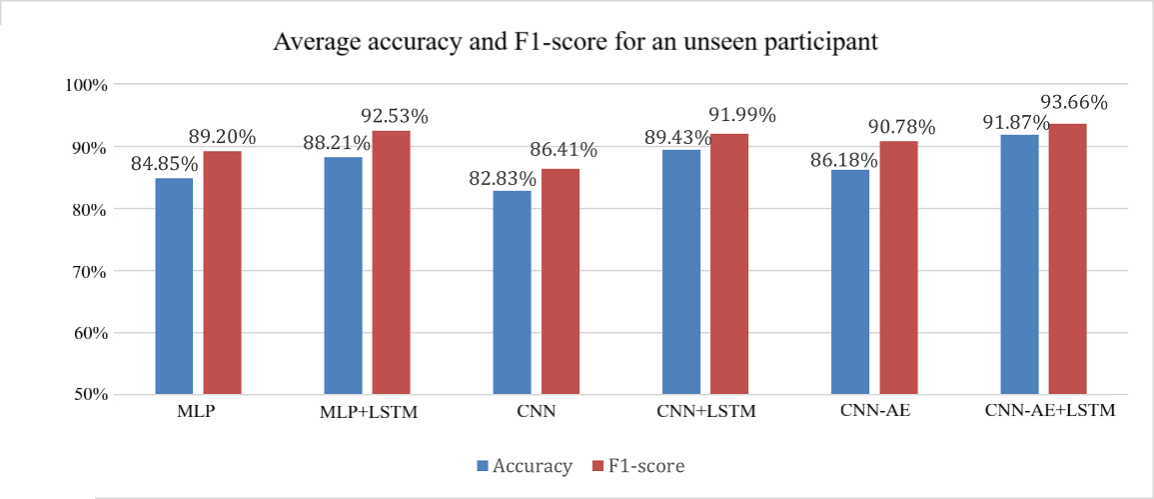
These are the resulting accuracies and *F*_1_-scores from the patient-wise classification. Each value is the overall average accuracy achieved on a patient never seen by the model. CNN: convolutional neural network; CNN-AE: convolutional autoencoder; LSTM: long short-term memory; MLP: multilayer perceptron.

Knowing that the CNN-AE+LSTM model functions best for processing timeseries data and that our motor task works is very important information, but it is also critical to evaluate how well the models would predict on a participant they had never seen before. In order to test this, a strict patient-wise training or testing data split was implemented [24]. The methodology used to split the data was simple; one of the 41 participants was selected at random for testing, and their trials were excluded from the training data. The models were trained until the training accuracy was above 95% accuracy, and once this limit was reached, the testing accuracy of the given participant’s trials was determined and then saved for later averaging. This was then repeated for each of the 41 participants, and the final resulting accuracy was calculated as the overall average of each saved testing accuracies from each participant. By averaging all possible testing conditions, the patient-wise method allowed us to interpret how well each of the models would work, as a true reflection of how likely a given model will perform correctly on an unseen participant. The precision and recall scores were also averaged in this way, allowing us to calculate the *F*_1_-score. It is important to note that it was not possible to balance the dataset for this particular test, which is reflected in Figure 7 by the worse performance of all non-LSTM models and by the slightly higher *F*_1_-scores relative to accuracy.

The primary result of this patient-wise evaluation is the importance of the LSTM layers. As can be seen in Figure 7, using LSTM layers seems to improve the model accuracy by roughly ~4% for all cases, with a similar effect on the *F*_1_-scores. These results once again confirm that the CNN-AE model is one of the best options for predicting children with ASD.

## Discussion

### Principal Findings

This study evaluated the effectiveness of various small-scale deep learning approaches for classifying children with and without ASD using IMU hand tracking data. Our results demonstrated that the best performance was achieved with a CNN-AE model combined with LSTM layers, yielding accuracies around 91% and *F*_1_-scores of 90.02% and 93.66%, respectively, using the k-fold and patient-wise evaluation methods. When it comes to the task of scanning sequence movement data for characteristic features of children with ASD, it seems clear that both of the CNN models overfit the data and perform worse than the MLP models, with the CNN-AE+LSTM model continuing to be the best classification option.

Additionally, it is important to understand why these models performed at the level that they did; this understanding could benefit future work involving small-scale models and medical data. Deep learning networks such as these are powerful tools, but they need to be tailored to their specific use cases. Normally, it is unusual to see a CNN model performing significantly worse than the MLP model; however, there could be several reasons for this. One likely reason is the data format: CNN models cannot interpret sequential data in the same way an MLP might be able to. It all comes down to where the optimization happens in each model type. In a CNN-type model, all of primary optimization happens inside the kernels, and a fixed-size filter then scans over an entire signal and looks for a specific set of features that the internal weights are optimized to find. Because these kernels are a fixed size, a single CNN layer can only ever look at a small chunk of the overall data at any given time to look for patterns. All of this to say, a CNN can struggle to capture long-range dependencies and complex patterns in sequence data. The convolutions performed by a CNN are better suited for locating features with short-range dependencies, or in other words, a CNN is better for detecting localized patterns. On the other hand, MLP type models are capable of learning complex, nonlinear relationships between the extracted features and the target variable. This is because every single neuron in an MLP layer is connected to every other neuron with an optimizable weight, meaning the entire input sequence can be evaluated at the same time. This flexibility can sometimes lead to a better performance in learning classifications from intricate, long, and complex patterns. Furthermore, while a CNN-AE model might only have CNN layers for detecting short-range patterns, it has the added bonus of compressing the input sequence into a lower-dimensional latent space, capturing essential features while filtering out noise. This results in a more efficient latent representation of the data, compared to the results of a CNN alone. Effectively, this means that the CNN layers of the autoencoder model will detect and significantly compress all possible information from short-range dependencies, while still preserving the necessary long-range dependencies to allow for accurate reconstruction. This highly compressed version of the input data can seriously mitigate overfitting, which likely explains why the CNN-AE models outperformed the regular MLP and CNN models. It is important to note that we did some experimenting with dropout layers, normally used to mitigate overfitting in simpler model types, but the results were mixed. The models became inconsistent, sometimes performing slightly better, sometimes performing worse. This is likely due to the small size of the dataset; future work with larger datasets should not ignore the possibility that dropouts could increase accuracy even further.

We can also see that despite the feature compression of the CNN-AE model and the ability for an MLP to identify some long-range dependencies, these models still do not perform very well without the addition of LSTM layers. These LSTM layers are critical to improved performance, increasing the accuracy for every type of model. This makes sense, as LSTM layers have a few major advantages for analyzing and learning from sequence type data. LSTM layers are very successful at learning long-range dependencies due to their internal memory, which can hold critical data. This internal memory leverages additive and subtractive gates, known as the input gate and the forget gate, respectively. These gates are mathematical functions that can learn to store useful information in the internal memory while discarding irrelevant information. Also, LSTM layers can handle sequences of different lengths, which is crucial for this project because each trial from each participant is a different length of time, ranging from roughly 10 to 40 seconds. Unlike the CNN and MLP models, which required a fixed input size of 4000×12 (accomplished with zero-padding), all of the LSTM layers were able to utilize that data of different input lengths, and all zero-padding on the data was removed before being input into the LSTM during training and testing. This likely improved accuracy even more because the layers were not forced to handle meaningless data in the form of zeros.

It also seems that the CNN-AE model did somewhat better during the patient-wise test compared to the cross-validation test. This might be explained by the slightly larger amount of training data it had access to; during cross-validation, 10% of the dataset was always excluded for testing, whereas with the patient-wise test, excluding 1 participant is closer to excluding ~1%‐2% of the dataset. This may also explain why the CNN+LSTM model did better during the patient-wise testing, as the larger dataset may have helped the model avoid overfitting. Overall, these are all critical factors to consider in the future when designing a deep learning model that will use medical or cognitive data. Proper fine-tuning of a model can allow for significantly less resource usage and better performance overall.

### Comparison to Prior Work

It is also worth noting that in our previous paper [20], we broke down our raw data into a set of useful statistics, such as reaction time, total displacement, average and max velocity, time to peak velocity, and more. We used 1-way ANOVAs to determine that children with ASD had significant differences in reaction time, velocity, acceleration, and total displacement. Compared to TD children, the children with ASD had a slightly longer average reaction time and significantly higher acceleration values, leading to a higher peak velocity. This corresponds to the children with ASD having an increased number of movement subunits (micro-adjustments in path during the reaching motions). Finally, children with ASD had a significantly higher total displacement during the task. Using this information, we have been able to conclude that the hardest motion patterns for our model to detect were the ones with the most similarity to TD children, with smoother movements and less movement subunits, more consistent accelerations, and a slightly slower peak velocity. Trials where a child with ASD might have really focused and avoided a slower reaction time or finished with a normal total displacement tended to make classification harder. Equally, cases with TD children where they might have been distracted, leading to increased movement jerkiness (increased movement subunits), also caused classification issues. It also seems likely that having each child perform the task multiple times gave the children time to learn and get better at the task, bringing the TD and ASD classes a bit closer together and harder to discriminate between. The consideration of these specific movement features is important for the future design of a physical motor task to better exemplify the differences between ASD and TD goal-based movement.

### Limitations

First, an important limitation of this study is the lack of a larger dataset containing a more diverse group of children. With only 41 participants, our model is unlikely to generalize well enough to function as a true diagnostic measure; it does, however, show the need for more robust research. Only one-third of our trials were of TD children; the rest were of individuals with ASD, which we attempted to mitigate by using specialized data balancing techniques to ensure that they would reach a generalization. These models would likely work significantly better if we were able to gather data from more TD children to ensure the true range of neurodivergence is recognized by the model. Increasing the size of the dataset would certainly increase its capability to generalize to new patients and likely reduce the number of false-positive and false-negative results. An even class distribution also eliminates the need for either data augmentation or data balancing methods, which can decrease the complexity of models and reduce the possibility for the introduction of errors and data leakage. Moreover, we did not exclude children with comorbid developmental disorders from the ASD group. While this approach enhances the ecological validity of our findings, it limits our ability to identify movement patterns specific to children with ASD. Future studies that will include participants with varying diagnoses are needed to further explore differences in movement characteristics between children with ASD and those with other developmental conditions.

Second, another limitation of this study was the equipment used to train and test these models. We were limited by run time and VRAM limits when attempting to test more complex networks. It is possible a larger LSTM section attached to the other models could achieve even better results. It might even be possible to build an LSTM-only model that rivals the ones built in this study, but it would require more powerful hardware to prototype a model like this. This is also true of a similarly built transformer classifier, where a transformer built to the limitations of our equipment was unable to perform with significant accuracy, but a much larger transformer might be able to achieve the same or better results. This limitation can also be seen as an advantage, as the small-scale custom models like the CNN-AE developed here require a significantly smaller amount of VRAM. This is a critical consideration as graphics processing unit prices continue to rise; access to powerful AI does not need to be gated by unnecessary costs for larger models that are not significantly more effective than cleverly designed small models.

Third, one final limitation worth considering is the lack of a comprehensive ablation study on these models. We attempted to tackle this problem in Table 1, which represents a fairly rigorous examination of general model hyperparameters such as layer count and neuron count. Unfortunately, this work falls short as it does not specifically validate other choices made such as the use of LeakyRelu activation functions, among other choices. Future work should consider performing an ablation study on these models and the data preprocessing steps to help break down which components and parameters of the models are critical for high accuracy results. Primary targets for the ablation study should be testing the effectiveness of the *z* scoring and min-maxing steps during normalization, as well as altering the number of points used in the moving average. Also worth testing is the number of batch normalization layers; the model may only need 1 or 2 of these layers, rather than the current model setups where every single layer is followed by a normalization. Overall, based on the testing performed during the prototyping phase, we are reasonably confident that the changes mentioned here would have only a minor effect on outcome accuracy.

### Future Directions

It is important to consider the context in which models like these might be used to aid the diagnosis of children with ASD. Considering the possible harm of false-positive and false-negative results is a top priority; a false-positive result could lead to unnecessary spending by parents and self-doubt among children diagnosed, while a false negative could lead to a child missing out on critical care during the most important period of their life. For these reasons, a high *F*_1_-score of 90.02% is another critical metric that shows our models are very effective, showing that the CNN-AE+LSTM model can avoid generating false-positive and false-negative results in most scenarios. For future work, there are some other important methods that could mitigate the effects of a false-positive diagnosis. This kind of diagnostic AI should be combined with the same behavioral interviews that children go through now (where the doctor performing the interview should not know the diagnosis provided by AI to avoid bias). In addition, continuing research should focus on using similar models to classify the severity of ASD rather than just producing a diagnosis. This could further limit the problems caused by a false-positive result, allowing doctors to correctly interpret the model results during the many cases when a diagnostic AI does not have a proper yes or no answer for a specific patient. ASD is a spectrum after all, so ensuring our models reflect this is a critical next step to this research. If the primary problems caused by a false-positive result can be mitigated in these ways, it may be possible to train our models to target and remove false-negative results, even at the expense of slightly more false-positive results that need to be caught by doctors during the process. Of course, the ideal goal would be to eliminate all false-positive results along with all false-negative results.

In the future, this research could be continued by collecting a larger dataset from a more diverse group of children with ASD and TD children, which would improve the robustness of the model. Additionally, further analysis could be performed on various kinematic variables, such as submovement stops, an atypical movement characteristic often observed in children with ASD [25,26]. This would aid in developing the most effective motor task for diagnosing children with ASD. Future work should also look at alternative methods of time series data analysis; clever techniques like using the Time Series Feature Extraction Library might be able to provide significant improvements to the LSTM portion of our models. Finally, future studies could explore the use of deep learning approaches to classify children with and without ASD based on additional data such as behavioral data (eg, eye tracking), or even physiological data (eg, heart rate and blood pressure) using small noninvasive recording devices.

### Conclusions

This study evaluated the effectiveness of several small-scale deep learning models for classifying children with and without ASD using IMU-based hand tracking data collected during a goal-directed motor task. Across all architectures tested, the CNN-AE+LSTM consistently achieved the highest performance, reaching an *F*_1_-score of 93.66% on unseen participant data. These results support the hypothesis that differences in motor behaviors between TD children and children with ASD can be detected using IMU-based hand tracking.

In contrast to the prevailing trend of retraining large-scale pretrained models, this work demonstrates that compact models with random initializations may also be effective for handling classification tasks involving goal-directed motor behavior.

## Acknowledgments

AI: artificial intelligence
ASD: autism spectrum disorder
CNN: convolutional neural network
CNN-AE: convolutional autoencoder
IMU: inertial measurement unit
LSTM: long short-term memory
MLP: multilayer perceptron
TD: typically developing
VRAM: video random-access memory
